# A Frailty Index Based On Deficit Accumulation Quantifies Mortality Risk in Humans and in Mice

**DOI:** 10.1038/srep43068

**Published:** 2017-02-21

**Authors:** K. Rockwood, J. M. Blodgett, O. Theou, M. H. Sun, H. A. Feridooni, A. Mitnitski, R. A. Rose, J. Godin, E. Gregson, S. E. Howlett

**Affiliations:** 1 Geriatric Medicine, Department of Medicine, Dalhousie University, Halifax, N.S., Canada; 2 Department of Pharmacology, Dalhousie University, Halifax, N.S., Canada; 3 Department of Physiology & Biophysics, Dalhousie University, Halifax, N.S., Canada

## Abstract

Although many common diseases occur mostly in old age, the impact of ageing itself on disease risk and expression often goes unevaluated. To consider the impact of ageing requires some useful means of measuring variability in health in animals of the same age. In humans, this variability has been quantified by counting age-related health deficits in a frailty index. Here we show the results of extending that approach to mice. Across the life course, many important features of deficit accumulation are present in both species. These include gradual rates of deficit accumulation (slope = 0.029 in humans; 0.036 in mice), a submaximal limit (0.54 in humans; 0.44 in mice), and a strong relationship to mortality (1.05 [1.04–1.05] in humans; 1.15 [1.12–1.18] in mice). Quantifying deficit accumulation in individual mice provides a powerful new tool that can facilitate translation of research on ageing, including in relation to disease.

In biological systems, mortality risk typically (if not universally)^1^ increases with age. Even so, that risk does not increase uniformly. In clinical medicine, *frailty*, the noun, refers to this heterogeneity in risk amongst people of the same age^2^. *Frail*, the adjective, is used to describe those (mostly older people) at increased risk^3^. Understanding frailty is important clinically, and not just in defining who is at greatest risk. Frailty also strongly influences what a given disease might look like. For example, in younger people or fit older adults, a heart attack is commonly signaled by chest pain. In frail older adults with a heart attack, chest pain is less common than are symptoms such as acute confusion, or being “generally unwell” or taking to bed^4^. The risk from frailty is thereby compounded by the adverse consequences of delay in diagnosis. Such considerations mean that understanding frailty is important in understanding age-related disease. Across the adult lifespan, the risk of most diseases increases with age, meaning that most diseases occur most often in older adults. Even so, for reasons of convention, convenience, cost and clarity of focus (or so it is claimed), most medical research is done on models that use young animals, which have not lived long enough to accumulate other health deficits^5^.

Under-representation of ageing is important because “the problems of old age come as a package”^5^,^6^. That package appears to influence both disease expression and disease risk. For example, in blood vessels, age-dependent cellular/tissue damage that goes unrepaired gives hypertension, a measurable (if asymptomatic) health deficit. Hypertension, in turn, increases the risk of clinically evident diseases such as chronic kidney injury, congestive heart failure and stroke. It is becoming clear that a range of subclinical and clinical age-related deficits, that are themselves not recognized as disease-specific risks, are associated with a greater chance of common age-related illness in older adults. One such example is the risk of dementia, which increases more in relation to the combined effects of several age-related health deficits (*e.g*. problems with teeth, feet, bowels) than it does in relation to such traditional risk factors as hypertension or diabetes^7^. Similarly, the risk of osteoporotic fracture is more closely related to such age-related health deficits (known as “non-traditional risk factors”) than it is to traditional risk factors such as body mass index and bone mineral density^8^. The same is true for non-traditional risk factors associated with coronary heart disease hospitalization and death^9^. Together these results suggest that seemingly unrelated items which manifest as age-related health deficits might reflect more widespread processes of ageing, which, as they accumulate, influence late-life illness^5^,^10^. This is why it is of some consequence that the problems of old age do not travel alone: to understand disease risk and expression, we must consider otherwise unmeasured heterogeneity in the health status of the organism/people so affected^10^.

To reduce unmeasured heterogeneity, we quantify how the package of age-related deficits influences the overall state of health. In humans we pioneered the concept of a “frailty index”. In a single variable, it measures a wide range of age-related health deficits (*e.g.* symptoms, signs, laboratory abnormalities)^11^,^12^,^13^. With a frailty index, the *actual number of health deficits* in an individual is counted and divided by the total number of *potential health deficits that were considered*, to yield a number between 0 (no deficits) and 1 (all possible deficits). For example, where 50 health deficits were considered (as with a health record or epidemiological survey), the frailty index score of someone with 5 deficits would be 5/50 = 0.10. Remarkably, even when differing health deficits, and differing numbers of health deficits are considered, their age-related accumulation shows significant stochastic dynamics, which for populations give characteristic rates of increase across the life course^12^,^14^. Further, people with high frailty index scores are at greatly increased risk of adverse outcomes, including death^12^. The force of mortality in relation to deficit accumulation gives a limit to frailty, such that the highest value of the frailty index score is not the arithmetic limit of 1. Instead, people with high frailty scores cannot survive additional health deficits; in almost every report, >99% of observed frailty index scores are <0.7^15^,^16^. In these ways, a frailty index based on deficit accumulation allows ageing itself to be investigated^17^. It can aid understanding for both specific age-related processes (*e.g*. cellular mechanisms related to myocyte hypertrophy^18^ and at larger scales, such as the health span response to calorie restriction or resveratrol^19^ or the network dynamics of ageing^20^).

Here, to evaluate whether quantifying deficit accumulation in humans can be translated to mice, we first replicated a frailty index analysis across the life course in humans. We then translated a life course frailty index in a naturally ageing colony of a common mouse model, the C57BL/6 strain, which we followed to extinction. We demonstrate similar properties of the frailty index in both humans and mice, including a characteristic distribution, similar values at similar life stages, a dose-response relationship with mortality and a submaximal limit. These properties arise even though individual deficits vary in how they accumulate. Together, these properties of age-related deficit accumulation allow a better way to understand disease mechanisms in the context of the overall extent of ageing in given subjects, including through the use of powerful genetic interventions. The approach can also improve the cost-effectiveness of studying ageing animals, thereby facilitating translational research across a range of illnesses.

## Results

### Individual deficits accumulated variably in both humans and mice

**Figure 1:**
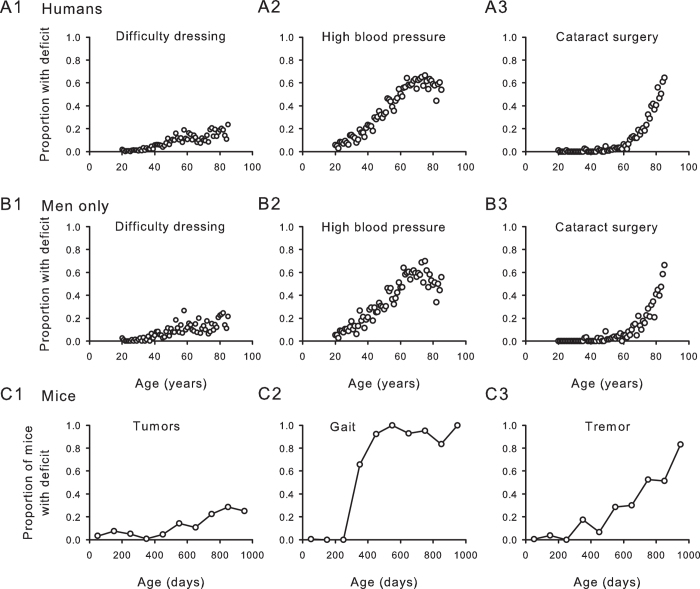
Individual deficits accumulate at varying rates in people and in mice. (**A**) Mean scores from pooled data from men and women show examples of individual deficits that exhibit different patterns of accumulation with age (n = 9169 people). Difficulty dressing (panel 1), high blood pressure (panel 2) and cataract surgery (panel 3) accumulated at different rates over time. (**B**, **panels** 1, 2, 3) Rates of deficit accumulation were similar for men only when compared to the sample shown in A (n = 4383 men). Data were binned and averaged each year for A and B. (**C, panels** 1, 2, 3) Tumours (panel 1), gait (panel 2) and tremor (panel 3) are shown as examples of deficits that demonstrate different progressions with age in male mice. Longitudinal data for each deficit were obtained from the entire cohort of mice and binned in 100 day increments (n = 251 mice).

Individual age-related deficits accumulated variably in humans of both sexes (Fig. 1A, upper panels 1–3), men only (Fig. 1B, middle panels 1–3) and in male mice (Fig. 1C, lower panels 1–3). Some deficits increased slowly at a near-constant rate (panels 1A1, 1B1, 1C1). Other deficits showed negative curvature (Panels 1A2, 1B2 and 1C2) or upward curvature (Panels 1A3, 1B3 and 1C3).

### In humans and in mice the mean frailty index increased with age

In humans, the mean value of the frailty index increased from 0.08 ± 0.07 in younger people (20–44 years) to 0.16 ± 0.11 in the middle-aged (45–64 years) to 0.24 ± 0.13 in those aged 65 + years (Table 1). For men, the corresponding numbers were younger = 0.07 ± 0.06; middle aged = 0.14 ± 0.11 and; older = 0.23 ± 0.12. In mice, the mean for the whole set of observations (n = 1074) increased from 0.05 ± 0.05 in the younger mice (30–299 days) to 0.21 ± 0.04 in the middle-aged mice (300–599 days) to 0.29 ± 0.07 in the older mice (600 + days).

**Figure 2:**
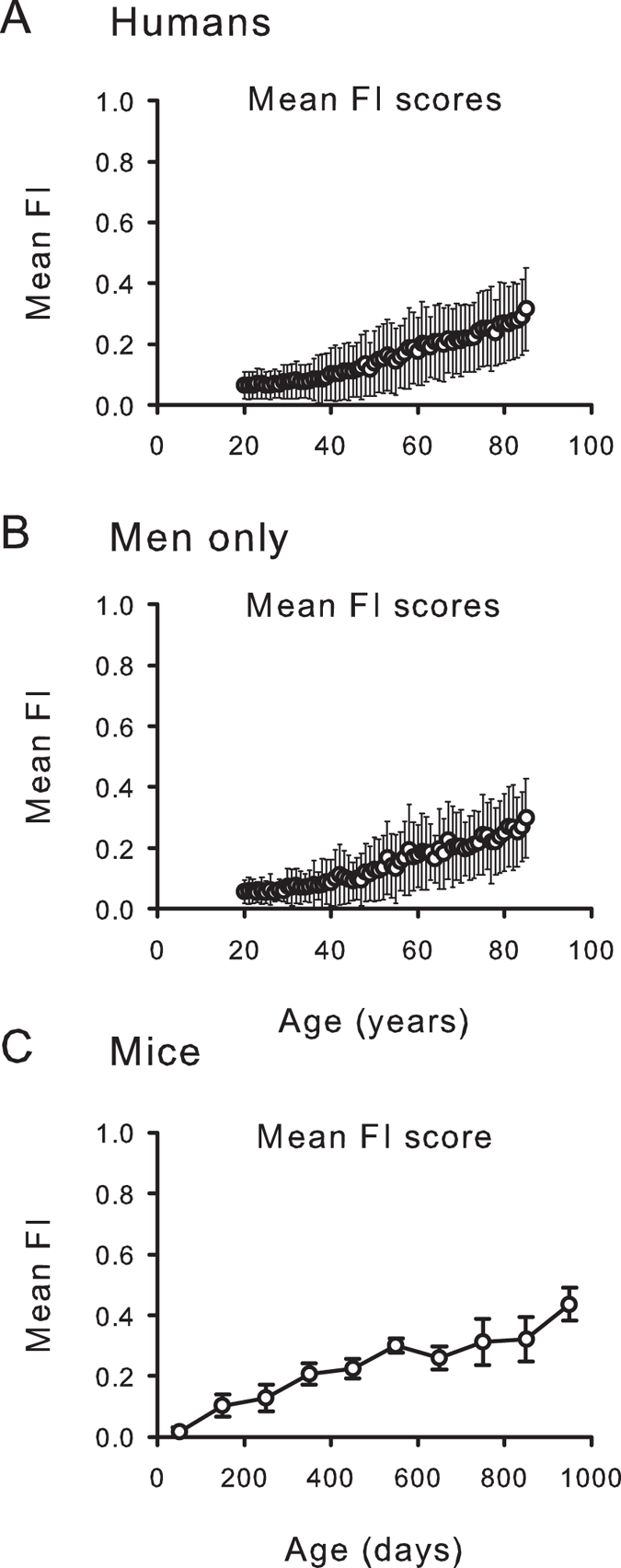
Mean ± SD frailty index (FI) scores increase at similar rates in people and in mice. (**A**) Data from 46 individual deficits were used to calculate a FI score for each person. FI scores increased with age in the pooled sample of men and women (n = 9169). Data were binned in one year increments and FI scores were averaged at each age. (**B**) Similar results were seen in men only, using the same increments, averages and binning (n = 4383). (**C**) An FI score was calculated from 31 individual mouse deficits for all 251 mice. Data were binned in 100 day increments and FI scores were averaged for each age group (±SD). In each panel, the essential idea of frailty – unmeasured heterogeneity in the health status of organisms of the same age – is evident in the standard deviations. Even so, the fits to a linear model for the mean values were r^2^ = 0.97 for humans, 0.96 for men only and 0.93 for mice. The fits to an exponential model were r^2^ = 0.98 for humans, 0.96 for men only and 0.87 for mice. All the fits were significant for p < 0.0001.

The mean value (±standard deviation) of the frailty index for all observations in humans (n = 9179) was 0.15 ± 0.12; for men (n = 4405), the mean frailty index was 0.14 ± 0.12 (Fig. 2A,B). For mice, for all observations (n = 1032), the mean frailty index was 0.16 ± 0.12. The mean frailty index increased across the life course (Fig. 2C). The slope of the natural logarithm of the frailty index versus age, which has been shown to correspond to the rate of deficit accumulation in animals and in people^12^, was 0.029 in humans (0.029 in men) and 0.036 in mice.

### In humans and mice the frailty index distribution broadened with age

**Figure 3:**
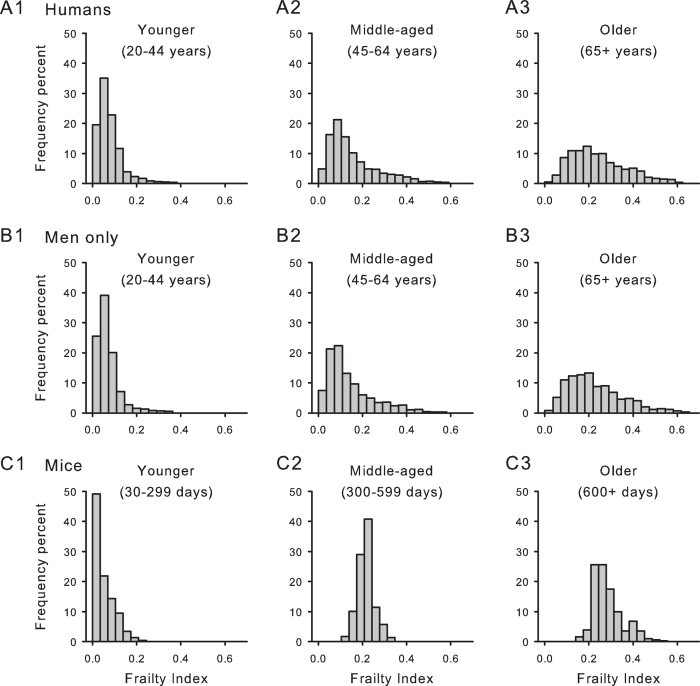
Mean frailty index (FI) scores increased and their distribution broadened with age in humans and in mice. **(A)** Frequency distributions of FI scores for younger (20–44 years; panel 1), middle-aged (45–64 years; panel 2) and older people (65 + years; panel 3) are illustrated. The frailty distribution moved right and broadened with age. (**B, panels** 1, 2, 3) Results were similar in men only when compared to the pooled sample shown in A. (**C, panels** 1, 2, 3) Frequency distributions of the FI scores for male mice also shifted to the right and broadened with increasing age. Data were stratified by age into three groups; younger (30–299 days old, panel 1), middle-aged (300–599 days old, panel 2) and old mice (600+ days old, panel 3).

The ranges differed only a little by age. In humans, the younger subjects’ frailty index range was 0.00–0.65 (Fig. 3, Panel A1), compared with 0–0.68 in the middle aged (Fig. 3, Panel A2) and 0.01–0.77 in the older group (Fig. 3, Panel A3). In men, the corresponding ranges were 0.00–0.55 (younger; Fig. 3, Panel B1); 0.00–0.59 (middle-aged; Fig. 3, Panel B2) and 0.03–0.77 (older; Fig. 3, Panel B3). The ranges in mice were 0.00–0.22 (younger; Fig. 3, Panel C1); 0.13–0.32 (middle-aged; Fig. 3, Panel C2) and 0.16–0.55 (older; Fig. 3, Panel C3).

Even though the absolute range differed little with age, the 99% limit (*i.e*. the highest frailty index scores that encompassed 99% of the observations) showed a noticeable difference across the life course. For the younger groups, the 99% limit was lowest (humans = 0.35; men only = 0.34; mice = 0.18). The middle-aged groups showed the next largest (humans = 0.51; men only = 0.49; mice = 0.31). The largest was seen in the older groups (humans = 0.59; men only = 0.58; mice = 0.49). In short, the distribution of frailty index scores broadened in all groups with age (Fig. 3).

### Greater frailty shortens survival in mice and humans

**Figure 4:**
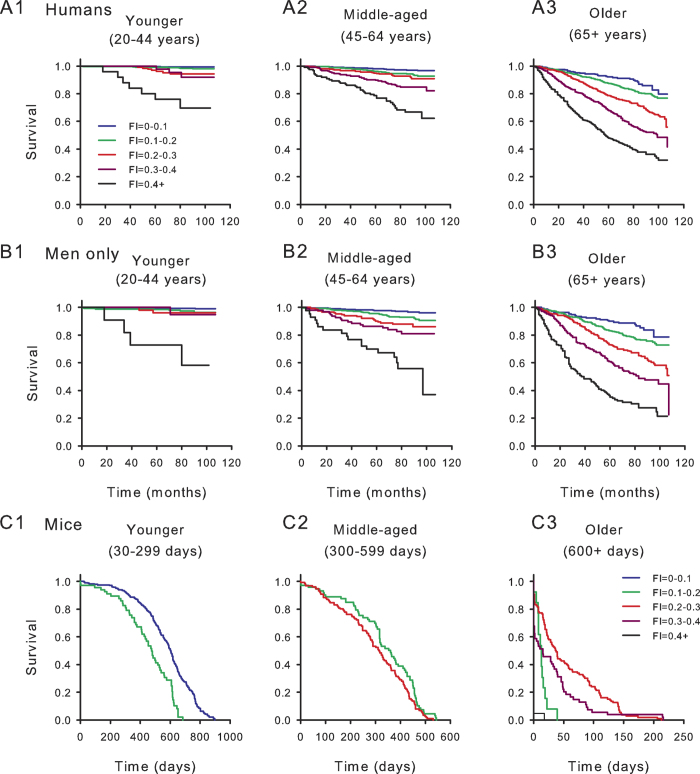
High frailty index (FI) scores predict mortality at all ages both in humans and in mice. (**A**) Kaplan Meier survival curves stratified by 0.1 increments of the FI for younger (20–44 years; panel A1, n = 4083), middle-aged (45–64 years; panel A2, n = 2642) and older people (65+ years; panel A3, n = 2444) are illustrated. At any age, higher frailty scores were associated with reduced survival. (**B**) Results were similar for men, (younger, Panel B1, n = 1867, middle-aged, Panel B2, n = 1292, older, Panel B3, n = 1242) although mortality was higher in men than in the whole human sample. (**C**) Kaplan Meier survival curves were also constructed for younger (30–299 days; panel C1, n = 251), middle-aged (300–599 days; panel C2, n = 229) and older (600+ days; panel C3, n = 158) mice. Each mouse is represented only once in each age stratum. In general, as FI scores increased survival declined in all three age groups of mice. Mortality was high in one FI stratum in the older group (*e.g*. FI = 0.1–0.2). This reflected a small number of very old mice with low FI scores and severe deficits that required immediate euthanasia.

Higher frailty index scores were associated with reduced survival in all groups (Fig. 4; Table 2). Deficits were less lethal in women than in men, so that the survival for the whole human sample (Fig. 4A, Table 2) was greater than for men (Fig. 4B). Deficits were most lethal in mice (Fig. 4C). For all groups, more information was provided by frailty for older subjects (Fig. 4A,B,C panels 2,3) than for the youngest ones (Fig. 4A,B,C panel 1). The hazard ratios for mouse survival were higher for any 0.01 frailty index increment than they were for the human samples (Table 2).

## Discussion

If research on the diseases of ageing is lagging by neglecting the other problems that come with age^5^,^6^, any remedy requires that those other problems be quantified^17^,^21^,^22^. Here we have shown that such a quantitative measure, a clinical frailty index based on health deficit accumulation, can be translated into mice and used to follow them across the life course; this last was endorsed as greatly preferable in a recent multidisciplinary report^21^. The mouse frailty index showed several essential features seen with human ageing. Although individual deficit accumulation patterns varied, the distribution of the frailty index broadened with age and mean deficit accumulation increased. At all ages, greater deficit accumulation was associated with reduced survival in age-adjusted models. The frailty index approach offers a probe by which the impact of individual problems can be understood in the context not just of chronological ageing, but of the increased hazard that goes with greater deficit accumulation^23^. The approach has been extended to demonstrate that, across a spectrum from subcellular to clinically detectable changes, frailty correlates with the degree of myocyte hypertrophy and contractile dysfunction^18^ as well as sinoatrial node dysfunction, myocardial interstitial fibrosis and altered expression of matrix metalloproteinases^24^ in the ageing heart. Given the potential repurposing of existing, approved medications such as rapamycin and metformin^25^, and pending the development of a comprehensive system of ageing biomarkers^26^, it also is a ready-made candidate for understanding how interventions to modify aspects of ageing processes might be evaluated^27^. A parallel approach and terminology can also facilitate clinician – researcher interactions, the relative lack of which is seen as an important barrier in translational research in ageing^28^.

Employing a frailty index can facilitate insights into how ageing works. Here, even amongst mice of similar genetic backgrounds, raised under similar circumstances, the level of deficit accumulation varied. Reflected in the broadening of the distribution of the frailty index with age, the stochasticity of frailty dynamics is significant as deficits accumulate^20^. Given that some degree of deficit accumulation is reversible^29^, understanding factors that allow deficits to decrease, even in old age, is important.

A 2016 report showed that the increase in deficit accumulation can be modelled solely on the basis of deficit interaction, without invoking time-dependent damage^20^ as has classically been done in studying age and mortality^30^,^31^. Here, deficits were both relatively higher and more lethal in mice, suggesting that their interactions are more damaging. If we accept that the accumulation of deficits reflects damage that goes unremoved or unrepaired^20^,^29^ then it follows that, even in an animal care facility environment in which the animals are relatively protected from predation, greater levels and lethality of age-related deficits likely reflect differences in investment in repair between mice and people.

A frailty index is especially useful in increasing the amount of data that can be collected on ageing animals. The high cost of ageing research reflects in part that unexpected death results in no data being available on that animal. In contrast, tracking frailty scores can allow at least some features of age-related change associated with specific conditions to be investigated, even when animals die ahead of planned experimental evaluation. Where death occurs before an experiment can be conducted, frailty index data can be compared with necropsy findings.

Our data must be interpreted with caution. The human data included men and women. The mouse data are for males only. We recognize the great virtue in studying ageing and frailty in female animals^32^; some data collection with female mice is ongoing and we plan to collect more. We also lack data on very frail mice (*e.g.* the 99% frailty index limit was 0.44) compared with very frail people. This reflects good animal husbandry practices. Indeed, we were intrigued that the veterinarians and animal care attendants who observed the mice, but who did not score a frailty index, the animals which they identified as needing to be sacrificed due to age-related debility had frailty index scores between 0.50–0.54. Tracking changes in the degree of deficit accumulation might provide some remedy against unexpected death and loss of animals before planned experiments. The designs of the two data sources are also different. The mouse data represent a small cohort followed to extinction, whereas we have only cross-sectional data with necessarily time-limited mortality follow-up on the humans, albeit with a much larger sample size. We attempted to mitigate the small mouse sample size by multiple observations. We were careful not to double-count mice in the same age range.

Translation here was from human studies that then were employed in mice. “Back translation” is now under way. The version of the mouse frailty index used here was modelled on a comprehensive geriatric assessment, and is suitable for longitudinal use. In contrast, the initial mouse frailty index chiefly used laboratory test data, arising in the context of terminal experiments^18^. More recently, we have developed a version of the frailty index for use in humans that employs only laboratory test data, as a means of understanding subclinical aspects of frailty^33^,^34^. Frailty is also now a target for treatment in and of itself^35^, arguing further for integrative methods for its measurement. For these reasons, combining the clinical and laboratory-based versions, in both humans and in mice, is motivating additional inquires by our group.

## Methods

### Study design and sample: mice

Two hundred fifty-seven male C57BL/6J mice were purchased from Charles River Laboratories (St. Constant, Quebec) at 3 to 4 weeks of age. Six mice died before any frailty assessments were completed, so these mice were excluded from the analysis for a final sample size of 251 mice (Table 1). Mice were housed in the Carleton Animal Care Facility in groups of three to five mice per microisolator cage and followed throughout their lives in a longitudinal study. They were kept on a 12-hour light/dark cycle and had free access to water and food (ProLab RMH 3500, Purina LabDiet, Aberfoyle, Ontario, Canada). Animal protocols followed the Canadian Council on Animal Care Guide to the Care and Use of Experimental Animals (CCAC, Ottawa, ON: Vol. 1, 2nd ed. 1993; Vol. 2, 1984) and were approved by the Dalhousie University Committee on Laboratory Animals. No randomisation was used. For all experiments, the investigator knew the age of the animal, but not its current or prior frailty scores.

### Study design and sample: humans

Secondary data for the human sample were acquired from the National Health and Nutrition Examination Survey (NHANES), a series of cross-sectional surveys investigating the health status of a nationally representative sample with examination, questionnaire and laboratory data^36^. Here, the 2003–2004 and 2005–2006 cohorts were amalgamated to give 20470 potential participants, of whom 10020 individuals were aged 20 and older. Of these 10020, we excluded 841 subjects due to missing frailty data and 10 additional subjects with missing mortality data for a final sample size of 9169 (Table 1). Criteria for a valid frailty score are detailed below. The NHANES survey protocol was approved by the National Center for Health Statistics Research Ethics Review Board of the Centers for Disease Control and Prevention, Protocol 98–12 for the 2003–2004 sample and Protocol 2005–2006 for the 2005–2006 cohort^37^. All participants provided written informed consent. All methods were performed in accordance with the Declaration of Helsinki regarding ethical standards for research involving human subjects.

### Data availability

Data were accessed through a public access data file available on the NHANES website http://www.cdc.gov/nchs/nhanes.htm^36^. Follow up mortality data were available using public-use linked mortality files, where mortality assessment was based on a probabilistic match between the NHANES records and the death certificate records of the National Death Index using available identifying information (i.e., Social Security Number, first name, middle initial, last name, month of birth, year of birth, day of birth, sex, father’s surname, state of birth, race, state of residence, marital status) (http://www.cdc.gov/nchs/ndi/index.htm)^38^. Time to death was calculated using person years of follow-up from the examination date until date of death or until the subject was censored on December 31st 2011. The datasets generated and/or analysed during the current study are available from the corresponding author on reasonable request.

### Frailty index construction in humans

The frailty index, as originally developed for use in humans, operationalizes frailty by dividing the number of deficits in an individual by the total number of deficits considered^39^. Here, the human frailty index comprised 46 deficits. All deficits were coded so that the presence of a deficit was scored as 1 and its absence was scored as 0. For variables with an intermediate response, a score of 0.5 was assigned. The frailty index was calculated by dividing the number of deficits present by the total number of deficits considered. For example, a subject with 25 of a possible 50 deficits would have a frailty index score of 25/50 = 0.5 A score of 0 represents full health, whereas a score of 1 represents a theoretical ‘complete’ frailty.

The human frailty index has been validated previously in the NHANES population^40^. A complete list of the deficits, procedures used and the coding guide has been published elsewhere^40^. Briefly, to be included in a frailty index, a deficit must meet the following criteria^39^: (1) it must increase with age; (2) it must be health-related; (3) it must be present in at least 1% of the study population; (4) it must not be present in 80% of the study population before age 80; (5) it must not be missing in more than 5% of the study population. Continuous variables were coded using cut-points proposed in the literature, as described in our previous work^34^. Empirically, >99% of people have frailty index scores <0.7^15^. A frailty score was only calculated for participants in whom <20% of the variables were missing. To calculate the frailty index in those missing fewer than 20% of variables, the denominator was reduced to reflect the number of available variables.

### Frailty index construction in mice

Our group has adapted this method to create a frailty index tool based on the accumulation of clinically apparent health deficits in naturally ageing mice^41^,^42^. A published checklist^41^,^42^ was used to evaluate 31 parameters that assessed deficits in the integument, musculoskeletal system, vestibulocochlear and auditory systems, ocular and nasal systems, digestive system, urogenital system, respiratory system and signs of discomfort. Mice were also weighed and body surface temperature measured with an infrared probe (Infrascan; La Crosse Technology). Body weight (g) and body surface temperature (°C) were scored based on deviation from the study average for that age and described previously^41^,^42^. Frailty was assessed at approximately 1, 6, 12 and 18 months and at varying ages thereafter. We also recorded the time to death for each mouse. Mortality occurred when animals either died suddenly or were euthanized due to illness.

### Statistical analysis

Statistical analyses were conducted using SPSS 20, R 3.2.4, MatLab and SigmaPlot. An alpha level of 0.05 was used to determine statistical significance. Demographic characteristics of the sample were expressed using mean frailty index scores ± SD, range (min and max) and 99th percentiles. The 99% limit was defined as the highest frailty index score that encompassed 99% of the observations. The association between age and the prevalence of selected individual deficits were graphed for mouse and human data. Density distribution curves were created for all frailty index scores. Curve estimation was used to assess the relationship between age and frailty score. To examine frailty across the life course, analyses were stratified by age group (20–44, 45–64, 65 + years of age for humans; 30–299, 300–599, 600+ days for mice). For the mouse frailty measurements, which were longitudinal, each mouse is represented only once in each age stratum, with the exception of the Cox regression described below. To investigate the classification and predictive ability of the frailty index score on mortality, we employed AUCs and age- and sex-adjusted Cox regression models. For the mouse data, frailty was treated as a time-dependent covariate in Cox regressions. Time was used as a time-variable in the Cox model. For these Cox regression models, power was high for both the mice data and the human data (1-beta > 0.95 at alpha < 0.05). Time intervals were created based on the time between the relevant frailty assessment and the death of each mouse. Obviously, each mouse death can be counted only once, but each mouse was assessed over their life span. The frailty index at the beginning of each age interval (young, middle-aged, old) was related to survival within that interval. Each hazard ratio represents the increased risk of mortality for every 0.01 increase in frailty index score. For the analysis by age group, a variable was calculated for each of the three age groups (*i.e*., 30–299, 300–599, 600+). Similar to a dummy variable, when the young group was the target, each mouse was represented by its frailty index value for time intervals ending at <300 days; values higher than this were scored as 0. Similarly, when the target was middle age, each mouse between 300 and 599 days was represented by its frailty index score, and was scored 0 otherwise. Likewise, the old variable had the corresponding frailty index values for intervals >600 days and was scored 0 otherwise. The three variables were included in a single Cox regression. Kaplan Meier survival curves demonstrated differences in mortality rates between frailty groups.

## Additional Information

**How to cite this article:** Rockwood, K. *et al*. A Frailty Index Based On Deficit Accumulation Quantifies Mortality Risk in Humans and in Mice. *Sci. Rep.* **7**, 43068; doi: 10.1038/srep43068 (2017).

**Publisher's note:** Springer Nature remains neutral with regard to jurisdictional claims in published maps and institutional affiliations.

## Figures and Tables

**Table 1:** Summary statistics for the human and mouse cohorts.

**HUMANS**	**Overall**	**Younger 20–44 years**	**Middle-aged 45–64 years**	**Older 65+ years**
**N**	9169	4083	2642	2444
**N Males**	4383	1867	1292	1242
**Mean FI**	0.15 ± 0.12	0.08 ± 0.07	0.16 ± 0.11	0.24 ± 0.13
**Range**	0–0.77	0.00–0.65	0.00–0.68	0.01–0.77
**99**^**th**^ **Percentile**	0.54	0.35	0.51	0.59
**MICE**	**Overall**	**Younger 30–299 days**	**Middle-aged 300–599 days**	**Older 600+ days**
**N**	251	251	229	158
**N Observations**	1032	495	228	309
**Mean FI**^**1**^	0.16 ± 0.12	0.05 ± 0.05	0.21 ± 0.04	0.29 ± 0.07
**Range**	0–0.55	0–0.22	0.13–0.32	0.16–0.55
**99**^**th**^ **Percentile**	0.44	0.18	0.31	0.49

^1^Values represent the mean ± SD.

**Table 2:** COX regression analysis of relationship between frailty and mortality for humans and for mice.

**Human - Age and Sex-Adjusted Model**				
	Full sample	Age group		
		20–44 years	45–64 years	65+ years
Frailty index	1.05	1.09	1.06	1.05
human (per 0.01)	(1.04–1.05)	(1.07–1.11)	(1.05–1.07)	(1.05–1.06)
**Mice - Time-Dependent Covariate Analysis**				
	Full sample	Age group		
		30–299 days	300–599 days	600+ days
Frailty index	1.15	1.27	1.07	1.17
mice (per 0.01)	(1.12–1.18)	(1.05–1.52)	(1.01–1.14)	(1.14–1.21)
